# Symmetry Dimension in Obsessive–Compulsive Disorder: Prevalence, Severity and Clinical Correlates

**DOI:** 10.3390/jcm10020274

**Published:** 2021-01-13

**Authors:** Aline P. Vellozo, Leonardo F. Fontenelle, Ricardo C. Torresan, Roseli G. Shavitt, Ygor A. Ferrão, Maria C. Rosário, Euripedes C. Miguel, Albina R. Torres

**Affiliations:** 1 Department of Neurology, Psychology and Psychiatry, Botucatu Medical School, Universidade Estadual Paulista—UNESP, Botucatu 18618-687, Brazil; alinepaesvellozo@gmail.com (A.P.V.); rictorresan@fmb.unesp.br (R.C.T.); albinatorres@gmail.com (A.R.T.); 2 Turner Institute for Brain and Mental Health, Monash University, Clayton, VIC 3168, Australia; 3 D’Or Institute for Research and Education & Institute of Psychiatry, Federal University of Rio de Janeiro, Rio de Janeiro 22290-140, Brazil; 4 Obsessive-Compulsive Spectrum Disorders Program, Department and Institute of Psychiatry, University of São Paulo, São Paulo 05403-010, Brazil; roseligshavitt@gmail.com (R.G.S.); ecmiguel@usp.br (E.C.M.); 5 Department of Psychiatry, Federal University of Health Sciences of Porto Alegre, Porto Alegre 90570-080, Brazil; ygoraf@gmail.com; 6 Department of Psychiatry, Universidade Federal de São Paulo, São Paulo 04038-000, Brazil; mariaceica.rosario@gmail.com

**Keywords:** obsessive–compulsive disorder, symptom dimensions, symmetry dimension, phenomenology, clinical features

## Abstract

**Background:** Obsessive–compulsive disorder (OCD) is a very heterogeneous condition that frequently includes symptoms of the “symmetry dimension” (i.e., obsessions and/or compulsions of symmetry, ordering, repetition, and counting), along with aggressive, sexual/religious, contamination/cleaning, and hoarding dimensions. **Methods:** This cross-sectional study aimed to investigate the prevalence, severity, and demographic and clinical correlates of the symmetry dimension among 1001 outpatients from the Brazilian Research Consortium on Obsessive–Compulsive Spectrum Disorders. The main assessment instruments used were the Dimensional Yale–Brown Obsessive–Compulsive Scale, the Yale–Brown Obsessive–Compulsive Scale, the USP-Sensory Phenomena Scale, the Beck Depression and Anxiety Inventories, the Brown Assessment of Beliefs Scale, and the Structured Clinical Interview for DSM-IV Axis I Disorders. Chi-square tests, Fisher’s exact tests, Student’s *t*-tests, and Mann–Whitney tests were used in the bivariate analyses to compare patients with and without symptoms of the symmetry dimension. Odds ratios (ORs) with confidence intervals and Cohen’s D were also calculated as effect size measures. Finally, a logistic regression was performed to control for confounders. **Results:** The symmetry dimension was highly prevalent (86.8%) in this large clinical sample and, in the logistic regression, it remained associated with earlier onset of obsessive–compulsive symptoms, insidious onset of compulsions, more severe depressive symptoms, and presence of sensory phenomena. **Conclusions:** A deeper knowledge about specific OCD dimensions is essential for a better understanding and management of this complex and multifaceted disorder.

## 1. Introduction

Despite having roots in normal phenomena [1,2], excessive preoccupations or preferences for symmetry and related behaviors may be a psychopathological manifestation, comprising the clinical picture of obsessive–compulsive disorder (OCD). In fact, repetitive behaviors related to symmetry and/or compulsions of ordering, arranging, repeating, and counting have long been reported among OCD sufferers [3]. OCD is an extremely heterogeneous psychiatric condition [4,5,6], and factorial analyses have consistently shown that such obsessions and compulsions constitute a separate dimension [6,7,8,9,10], along with aggressive, sexual/religious, contamination/cleaning, and hoarding dimensions. In this article, the dimension of symmetry, ordering, repeating, and counting will only be referred to as the “symmetry dimension”.

Important longitudinal studies [11,12] demonstrated that OCD dimensions present considerable stability over time, with most symptoms waxing and waning within specific dimensions rather than shifting between dimensions. Compulsions related to symmetry are also frequently observed in children and adolescents with OCD [13,14,15] and, in a Brazilian community study of students aged 14 to 17 years, the prevalence of the symmetry dimension was 85.3% [16]. The symmetry dimension was the most common (67.6%) among 139 Chinese OCD patients examined by Li et al. [17].

Functional neuroimaging studies suggest that OCD dimensions may be mediated by partially distinct subjacent neurocircuits [18,19], and that among them, the symmetry dimension may have unique genetic [20,21] and neural [18,19], correlates. In neuropsychological studies, higher scores in the symmetry dimension were associated with poorer verbal fluency, which involves set shifting capacity and cognitive flexibility [22,23,24].

Regarding demographic and clinical correlates, the symmetry dimension has been associated with male sex [4,17,25,26,27,28,29], early onset of obsessive–compulsive symptoms [8,27,28,30,31,32,33,34,35], longer duration of symptoms [21,27,36], and presence of tics or tic disorders [37,38]. Additionally, associations with family history of OCD [8,39,40,41,42], greater OCD severity and functional impairment [16,21,26,32], and suicidal behaviors [43,44] have been reported.

Another interesting clinical feature related to the symmetry dimension is that these patients usually present poorer insight [32,45] and, therefore, tend to seek less help for their mental health problem [46]. Moreover, the motivations underlying the compulsive rituals usually differ from those of other OCD dimensions, i.e., individuals with symmetry-related symptoms often do not report concomitant typical obsessions or fear specific consequences that are prevented by the repetitive behaviors, but rather exhibit a strong need for uniformity and an intense drive to alleviate feelings of incompleteness or imperfection, known as “sensory phenomena” [47,48,49].

In other words, symmetry compulsions are usually aimed at reducing feelings of discomfort, manifested as an unpleasant subjective experience that something is not right, and performing their behaviors (e.g., arranging behaviors) until things look just right [50,51,52,53]. Regarding psychiatric comorbidities, the symmetry dimension has been associated with tic disorders [37,38], attention deficit and hyperactivity disorder (ADHD) [41], panic disorder and agoraphobia [21,30] posttraumatic stress disorder (PTSD) [21], obsessive–compulsive personality disorder [8,21,54], substance use disorders [30,41], bulimia nervosa [15], and bipolar disorder [30].

Despite being a very common clinical manifestation of OCD, specific studies on the symmetry dimension are scarce and usually involve relatively small samples. Further, to our knowledge, no previous study has specifically explored all symmetry-related symptoms (e.g., symmetry, ordering, repeating, and counting) using a dimensional instrument of assessment. Therefore, the existing literature seems insufficient to inform clinicians on particular characteristics of patients presenting symmetry-related symptoms. The in-depth knowledge about the correlates of specific OCD dimensions is essential for a better understanding of this complex and multifaceted disorder and, consequently, may help guide clinical practice. The aim of this study was to estimate the prevalence, severity, and the demographic and clinical profiles of OCD patients with symptoms of the symmetry dimension in a large multicenter study of patients seen in tertiary and specialized services, comparing patients with and without this symptom dimension.

Our main hypotheses were that the majority of our patients would present symptoms of the symmetry dimension (due to the characteristics of the sample, including high clinical severity, early mean age at onset, and high comorbidity with tic disorders) and, according to the literature cited above, that it would be associated with male sex, early onset and higher severity of OCD, family history of OCS, sensory phenomena, suicidal behaviors, poorer insight, and comorbidity with tic disorders, ADHD, panic disorder and agoraphobia, substance use disorders, bulimia nervosa, PTSD, and bipolar disorder.

## 2. Material and Methods

### 2.1. Subjects

The sample consisted of 1001 OCD patients (955 adults and 46 children/adolescents) from the Brazilian Research Consortium on Obsessive–Compulsive Spectrum Disorders (C-TOC). Participants were recruited from seven Brazilian universities and interviewed between 2003 and 2009. The diagnosis of OCD was determined according to the DSM-IV criteria [55] and confirmed using the Structured Clinical Interview for DSM-IV Axis I disorders (SCID-I) [56]. Patients with psychotic and organic disorders or any other condition that could impair their understanding of the research protocol were excluded (*n* = 8). All patients were interviewed face-to-face by experienced and trained psychologists or psychiatrists. A complete description of the C-TOC implementation and methods has been previously published [57].

### 2.2. Assessment Instruments

The main assessment instruments that were used are briefly described below:

1. Yale–Brown Obsessive–Compulsive Scale—Y–BOCS [58,59]. This scale evaluates the severity of obsessive–compulsive symptoms (OCS) in the previous week, with 10 items ranging from 0 (no symptom) to 4 (extreme severity), and a maximum score of 40 (20 for obsessions and 20 for compulsions).
2. Dimensional Yale–Brown Obsessive–Compulsive Scale—DY–BOCS [60]. This scale has 88 items investigating the presence and severity of six OCD symptom dimensions: harm/aggression, sexual/religious, contamination/cleaning, symmetry, hoarding, and miscellaneous. The clinical severity of each dimension (both current and in the worst phase) is evaluated in terms of frequency, distress, and interference, with a maximum score of 15, five for each aspect. It also provides an assessment of overall severity of OCD, which ranges from 0 to 15 for symptoms and 0 to 15 for the impairment caused by them, with a total global score ranging from 0 to 30. Information on lifetime occurrence of the symmetry dimension (i.e., 12 symmetry, ordering, repeating, and counting symptoms) was obtained with the DY–BOCS checklist. This checklist was used to split the sample into two comparison groups: OCD with symmetry (OCD + Sym) and OCD without symmetry (OCD − Sym) dimension.
3. USP Sensory Phenomena Scale—USP-SPS [61]. This scale evaluates the presence and severity of sensory phenomena (SP) that precede or accompany compulsions. SP includes bodily sensations (usually tactile, muscular, or skeletal–visceral) and mental sensations (e.g., discomfort, “energy” that needs to be released, feeling of incompleteness, just not right perceptions or experiences). The current and past scores range from 0 to 15, with higher scores indicating more severe SP. This variable was analyzed both as categorical (presence/absence of SP) and quantitative (severity of SP).
4. OCD Natural History Questionnaire [62]. This detailed instrument was developed at Yale University to investigate OCS onset and clinical course. It is divided into three parts exploring (a) the onset of obsessions and compulsions, (b) the course of symptoms (with a comprehensive list of events or situations that may lead to their improvement or aggravation), and (c) the worst phase of symptoms.
5. Structured Clinical Interview for DSM-IV Axis I Disorders—patient edition—SCID I/P [56]. The SCID-I was used to evaluate comorbid conditions and to confirm the OCD diagnosis. It is a widely used semi-structured interview, considered as the “gold-standard” to evaluate Axis I psychiatric diagnoses, according to the DSM-IV criteria [55] In this study, additional modules for the diagnosis of tic disorders and several impulse control disorders were included.
6. Beck Depression Inventory—BDI [63]. This inventory has 21 items investigating symptoms and attitudes related to depressive disorders, whose intensity varies from 0 to 3 (maximum score: 63). This instrument was used to evaluate the severity of depressive symptoms in the previous week, as a quantitative variable, and also in categories: absent or minimal symptoms (score from 0 to 9), mild (10–18), moderate (19–29), and severe symptoms (30–63).
7. Beck Anxiety Inventory—BAI [64]. The BAI is used to evaluate the severity of anxiety symptoms and is also composed of 21 items scored between 0 and 3 (maximum: 63). Each item describes subjective, somatic, or panic-related anxiety symptoms in the previous week. The score was analyzed as a quantitative variable.
8. Brown Assessment of Beliefs Scale—BABS [65]. This scale evaluates different aspects of insight, including conviction about beliefs, perception of others’ views, explanation of different visions, rigidity of beliefs, attempts to refute them, ability to recognize a psychological cause for the beliefs, and ideas of reference. Each of the six items is rated from 0 to 4 (maximum score: 24), with higher scores indicating poorer insight. It also includes a final evaluation of the patient’s level of insight in five categories: excellent, good, moderate, poor, or absent.

Data on family psychiatric history were obtained with the patients using a questionnaire developed by the C-TOC, which includes the SCID-I screening questions. The questions regarding patient’s suicidal behavior (yes or no answers) were as follows: have you ever (1) thought life was not worth living, (2) wished to be dead, (3) thought about killing yourself, (4) made suicidal plans, (5) attempted suicide; as well as 6) do you have current suicidal thoughts?

### 2.3. Ethical Aspects

The Research Ethics Committee of Botucatu Medical School (UNESP) approved the research protocol on 5 June 2017 (doc. 2104036) after the concordance of the C-TOC local leaders of the University Hospitals involved. All participants signed a term of informed consent (parents or legal guardians of children and adolescents also signed the consent form). Patients were assured that if they decided not to participate in the study, this would not interfere with the continuity of treatment (38 refusals).

### 2.4. Statistical Analysis

All statistical analyses were performed using the STATA 12.0 software (Stata Corporation; Stata Statistical Software, Release 12.0 (2011); College Station, TX, USA). Initially, a descriptive analysis of the outcome and the explanatory variables was conducted: categorical variables were described as absolute (*n*) and relative (%) values, and continuous variables as means and standard deviations (SD) or medians (ranges). Then, patients with and without symptoms of the symmetry dimension were compared (bivariate analyses) using the Pearson’s chi-square or Fisher’s exact tests for categorical variables, and the Student *t*-test or Mann–Whitney tests for quantitative variables, according to the normality or not of the distributions (skewness kurtosis test for normality). As measures of effect size, odds ratios (ORs) with confidence intervals were calculated for qualitative variables and Cohen’s D for quantitative variables. Finally, a stepwise backwards logistic regression (LR) analysis was conducted, excluding each variable presenting the least significant association. All variables that were significant in the bivariate analysis (*p* < 0.05) were included in the LR model, except those that presented multicollinearity with others (variation inflation factor > 10). The variable “sex” was also included in the LR due to its importance in the literature. A standard value of *p* < 0.05 was adopted to reject the null hypothesis.

## 3. Results

The lifetime prevalence of symptoms of the symmetry dimension was 86.8% (*n* = 869). The most common symptoms within this dimension were obsessions about the need for things to be perfect or exact (*n* = 638; 73.4%), compulsions to check for errors (*n* = 577; 66.4%), re-reading or rewriting (*n* = 577; 66.4%), ordering or arranging rituals (*n* = 535; 61.6%), obsessions about symmetry (*n* = 472; 54.3%), the need for repeating routine activities (*n* = 461; 53.0%), fear of not saying things exactly correct (*n* = 437; 50.3%), counting compulsions (*n* = 336; 38.7%), avoidant behaviors to prevent obsessions or compulsions about symmetry or exactness (*n* = 314; 36.1%), compulsions involving symmetrical touch or symmetrical actions/movements (*n* = 297; 34.2%), other mental rituals besides checking or balancing (*n* = 282; 32.5%), and need to touch, rub, or tap (*n* = 249; 28.7%).

In the total sample, symmetry was the most severe dimension, with a median score of 10 (mean 8.76, SD 4.75) in the worst phase, followed by the contamination/cleaning (median 9; mean 7.62, SD 5.49), aggressive (median 8; mean 6.73, SD 5.56), sexual/religious (median 5, mean 5.68, SD 5.65), and hoarding dimensions (median 2, mean 3.89, SD 4.60). The current (previous week) severity of the symmetry dimension in the total sample was 8 (median), with a mean of 7.34, SD 4.64. Among patients with symmetry dimension, the mean scores for current and past symptoms were 8.43 (3.94) and 10.09 (3.54), respectively. Severity in the symmetry dimension correlated significantly with severity in all other dimensions.

The demographic characteristics of the participants are presented in Table 1 (bivariate analysis). Patients with symmetry dimension were younger and less likely to be married and to have children. The groups did not differ significantly regarding sex and other demographic features.

Table 2 presents the main clinical characteristics of the two study groups, including effect size measures (bivariate analysis). Patients with the symmetry dimension had earlier onset of obsessive–compulsive symptoms (OCSs), higher frequency of insidious onset of OCSs, lower age of symptoms interference, higher severity of OCSs (Y–BOCS and DY–BOCS scores) and of depressive symptoms (BDI score). When depressive symptoms were analyzed in categories, patients with and without the symmetry dimension also differed significantly (*p* = 0.039): minimal or absent (29.0% vs. 40.9%), mild (30.7% vs. 28.8%), moderate (25.1% vs. 18.9%), and severe symptoms (15.2% vs. 11.4%). In addition, the group with symmetry symptoms presented higher frequency and severity of sensory phenomena (USP-SPS), poorer insight (BABS score), and higher frequency of lifetime suicidal ideation and suicide attempts.

The findings of the bivariate analysis regarding lifetime comorbidities are presented in Table 3. When compared to the other patients, those with symmetry-related symptoms were more likely to present tic disorders, ADHD, major depressive disorder, and PTSD (Table 3).

Table 4 shows the final results of the logistic regression. The variables that remained associated with the symmetry dimension were earlier age at onset of OCD symptoms, insidious onset of compulsions, more severe depressive symptoms, and presence of sensory phenomena.

## 4. Discussion

This is the largest study so far exploring the prevalence and correlates of the symmetry dimension in patients with OCD. Its lifetime prevalence was 86.8%, and the clinical features independently associated with the symmetry dimension were lower age at onset of OCSs, insidious onset of compulsions, presence of sensory phenomena, and higher severity of depressive symptoms. This is a high prevalence, as most studies with OCD sufferers describe rates of symmetry, ordering, repeating, or counting symptoms to be between 30.9% and 75.8% [14,17,27,29,32,35,37,41,45,66,67]. This discrepancy may be partially explained by methodological differences, including sample composition (origin, gender, age range) and assessment instruments. Unlike most previous studies, we used an interview that has 12 items assessing symmetry-related obsessions and compulsions (DY–BOCS), twice the number (and therefore more comprehensive in terms of OCS) of items in the Y–BOCS checklist, which is the most used scale in the literature (see Table 5).

Besides using a detailed and dimensional instrument of assessment, the high prevalence of symmetry symptoms in our sample may also reflect its selection in specialized/tertiary services, which usually includes more severe cases (according to the CGI, 52.4% were severe or extremely severe). In addition, two other clinical parameters previously associated with symmetry-related symptoms were common in our sample: early age of symptom onset (mean: 12.6 years), and comorbidity with tic disorders (28.4%). In contrast, male sex was not associated with the symmetry dimension in our sample, unlike several studies [4,17,25,28,29] but in accordance with others in which men and women were equally distributed in this dimension [8,21]. Unmarried status was also associated with the symmetry dimension in two studies [21,32].

Obsessions or subjective feelings related to symmetry and/or compulsions of ordering, repeating, and counting have been consistently associated with earlier age at onset of OCS or OCD [8,27,28,30,31,32,33,34,35]. Such an association can be understood from a developmental perspective, and it is possible that childhood onset OCD represents a distinct, neurodevelopment form of the disorder [70,71]. In a previous study with adult patients [33], symptoms of the symmetry dimension presented the earliest age of onset of all, even after adjusting for gender and comorbidity with tic disorders, two factors consistently associated with early onset of OCD symptoms in the literature.

Regarding the type of symptom onset, insidious onset was more frequent in patients with symmetry-related symptoms, particularly compulsive rituals, manifestations that predominate in this dimension. This is a new finding that should be explored in future studies. Nevertheless, it may be a relevant clinical feature for health planning and care, since it indicates a possible opportunity for intervention before such symptoms aggravate, becoming clinically relevant.

Except for hoarding, there are few studies investigating the associations between specific OCD dimensions and psychiatric comorbidities. In our study, in the bivariate analysis, patients with symmetry-related symptoms were more likely to present comorbidity with major depression, PTSD, ADHD, and tic disorders. These comorbidities, except major depression, have been associated with the symmetry dimension in at least one study each [21,37,38,41]. In the present study, however, no comorbid disorder remained associated with the outcome in the logistic regression, only higher BDI score. More severe depressive symptoms may amplify the negative impact of OCD symptoms, worsen the insight regarding them, and, consequently, interfere with help-seeking and adherence to treatment [72,73]. However, this association was only marginally significant, and its clinical relevance should be confirmed or refuted in future studies. Potential investigations should examine prospectively the impact of symmetry-related symptoms in help-seeking behaviors, and also on adherence and response to treatment.

The presence of sensory phenomena (SP), however, seems to be a consistent clinical feature of patients with symptoms of the symmetry dimension. In fact, several studies described the association of symmetry symptoms and feelings of incompleteness or SP [47,49,51,52,53,74]. Sibrava et al. [49] examined the impact of the feeling/sensation/perception of incompleteness in 307 adults with OCD and reported that patients with clinically relevant incompleteness had higher OCD severity, more comorbidities, poorer quality of life, and higher rates of disability and unemployment, besides presenting more symptoms of the symmetry dimension. Notably, sensory phenomena and “tic-like” compulsions (i.e., need to “touch, tap or rub” or rituals involving “blinking or staring”), along with the symmetry dimension have been associated with the putative “early-onset” subtype of OCD in studies focusing on age at symptoms onset [75,76,77].

Besides sensory phenomena and “tic-like” compulsions, some studies described associations among symmetry–ordering–repeating–counting compulsions, early onset, and some variables that were significant in the bivariate analysis, such as being single [78], higher OCD severity [75,78,79,80], comorbidity with ADHD [76], and tic disorders [75,76,77]. On the other hand, variables that are frequently associated with early-onset OCD, particularly male sex [31,77,79,80,81] and family history of OCD [77,80,81], were not significant correlates of the symmetry dimension in the present study. Therefore, some features seem to partially overlap in OCD patients with the symmetry dimension and with early-onset of symptoms. Additionally, in an important review article on OCD subtypes by de Mathis et al. [82], male sex and “tic-like” compulsions related not only to early-onset OCD, but also to the putative “tic-related” phenotype, while symmetry-related symptoms, sensory phenomena, and ADHD were characteristics more closely associated with the former phenotype. Taken together, these findings suggest that OCD is indeed a mosaic of clinical manifestations that present partial phenomenological overlap and possibly also partially distinct etiological underpinnings.

Certain limitations should be considered when interpreting the results of this study. The sample consisted of OCD patients recruited in specialized services who may present greater clinical severity when compared to other clinical or community samples. Thus, the generalizability of the results may be somewhat limited, but they should be applicable to a large number of patients with moderate to severe OCD symptoms. The cross-sectional design explores the associations between dependent and independent variables, but not causal relations. Further, someone could argue that increased prevalence of symmetry, ordering, repeating, and counting symptoms could reflect counting or repeating in the context of other symptoms (e.g., patients who count while washing). However, we feel this possibility to be unlikely, as the DY–BOCS is able to differentiate primary symmetry-related symptoms from those occurring in other contexts. Finally, personality disorders and response to treatment were not evaluated. Some data were retrospective and, therefore, subject to recall bias.

Despite these limitations, the current study showed, in the largest sample assessed to date, that OCD patients with symmetry dimension symptoms have an earlier onset of OCS, more insidious onset of the compulsions, more severe depressive symptoms, and higher frequency of sensory phenomena. These findings reinforce the need for the early identification and treatment of these symptoms. Especially for children and adolescents, family members and teachers should be informed about these obsessions and compulsions, which are usually ego-syntonic and may be mistaken by normal child behaviors or personality traits (e.g., perfectionism). Being more insidious, such symptoms may lead to gradual family accommodation, delay in treatment seeking, and worse prognosis.

The early and insidious onset of symmetry-related symptoms represents a possible window of opportunity for preventive measures, in order to avoid aggravation and further interference of these symptoms in the individual’s functionality. Psycho-education of parents and teachers about symmetry–ordering–repeating–counting symptoms and related sensory phenomena may, therefore, favor early identification and treatment seeking. A deeper knowledge about the correlates of specific OCD dimensions is crucial for a better understanding of this complex and multifaceted disorder. Additionally, it may lead to more tailored therapeutic interventions and, hopefully, a better prognosis.

In conclusion, the results are consistent with some previous studies indicating a high prevalence and some distinctive clinical features of OCD patients with symptoms of the symmetry dimension. These patients had lower age at onset of OCS, more insidious onset of compulsions, and more sensory phenomena. Some of these clinical features have also been associated with the putative “early-onset” or “tic-related” subtypes of OCD, thus suggesting a partial overlap among these clinical profiles.

## Figures and Tables

**Table 1:** Demographic characteristics of 1001 OCD patients with (OCD + SYM) and without (OCD − SYM) symptoms of the symmetry dimension.

	Total *n* = 1001	OCD + SYM *n* = 869 (86.8%)	OCD − SYM *n* = 132 (13.2%)	*p*	OR (95% CI) or Cohen’s D *
Age (years)—mean (SD)	34.8 (13.0)	34.4 (12.9)	37.9 (13.2)	**0.003**	0.27 *
Sex (male)	432 (43.2%)	371 (42.7%)	61 (46.2%)	0.447	1.15 (0.80–1.67)
Marital status					
Single	544 (54.4%)	484 (55.7%)	60 (45.5%)	**0.028**	**1.51 (1.04–2.18)**
Married or cohabiting	377 (37.7%)	314 (36.1%)	63 (47.7%)	**0.010**	**0.62 (0.43–0.90)**
Has child(ren)	390 (39.0%)	323 (37.2%)	67 (50.8%)	**0.003**	**0.57 (0.40–0.83)**
Lives alone	73 (7.3%)	64 (7.4%)	9 (6.8%)	0.822	1.09 (0.53–2.24)
Education (years)—mean (SD)	14.6 (5.0)	14.6 (4.8)	14.3 (5.9)	0.591	0.06 *
Occupational status					
Unemployed	154 (15.4%)	135 (15.5%)	19 (14.4%)	0.735	1.09 (0.65–1.84)
Working	497 (49.8%)	422 (48.8%)	75 (56.8%)	0.083	0.72 (0.40–1.05)
Religious practice (any)	547 (54.7%)	473 (54.4%)	74 (56.1%)	0.726	0.94 (0.65–1.35)
Ethnicity (non-Caucasian)	169 (16.9%)	144 (16.6%)	25 (18.9%)	0.498	0.85 (0.53–1.36)
Social class **					
A/B (higher)	554 (55.3%)	474 (54.6%)	80 (60.6%)	0.192	1.28 (0.88–1.86)
C/D/E (lower)	447 (44.7%)	395 (45.5%)	52 (39.4%)		

Bold values represent the statistically significant results. Underlined variables were included in the logistic regression analysis; OCD: obsessive–compulsive disorder; SD: standard deviation; * Cohen’s D; ** based on sociodemographic criteria of the Brazilian Association of Market Research Institutes (ABIPEME).

**Table 2:** Clinical characteristics of 1001 OCD patients with (OCD + SYM) and without (OCD − SYM) symptoms of the symmetry dimension.

	Total *n* = 1001	OCD + SYM *n* = 86 (86.8%)	OCD − SYM *n* = 132 (13.2%)	*p*	Cohen’s D or OR (95% CI)
**Clinical Course**					
Age at OCS onset (years)—mean (SD)	12.6 (7.3)	11.8 (6.5)	17.4 (9.9)	**<0.001 ^e^**	**0.77**
Age at onset—obsessions (years)—mean (SD)	13.2 (8.0)	12.5 (7.3)	17.5 (10.5)	**<0.001 ^f^**	**0.63**
Age at onset—compulsions (years)—mean (SD)	13.0 (7.7)	12.2 (6.9)	18.2 (10.1)	**<0.001 ^e^**	**0.80**
Age at symptoms interference (years)—mean (SD)	21.8 (10.6)	21.3 (10.5)	24.5 (11.1)	**0.002 ^e^**	**0.30**
Age at first treatment (years)—mean (SD)	29.4 (12.9)	29.1 (12.9)	31.2 (13.2)	0.096 ^e^	0.16
**Clinical Severity**					
Y–BOCS—total score—mean (SD)	25.5 (7.5)	25.7 (7.3)	23.9 (8.8)	**0.010 ^e^**	**0.24**
Y–BOCS—obsession score—mean (SD)	12.7 (3.9)	12.8 (3.9)	12.2 (4.4)	0.115 ^e^	0.15
Y–BOCS—compulsion score—mean (SD	12.8 (4.1)	13.0 (4.0)	11.8 (5.0)	**0.002 ^e^**	**0.29**
DY–BOCS—current total score—mean (SD)	21.1 (6.3)	21.3 (6.1)	19.9 (7.0)	**0.030 ^f^**	**0.22**
DY–BOCS—worst phase total score—mean (SD)	23.6 (5.1)	23.8 (5.0)	22.8 (5.5)	0.082 f	0.20
BDI—total score—mean (SD)	16.4 (11.2)	16.8 (11.2)	14.2 (11.3)	**0.014 ^e^**	**0.23**
BAI—total score—mean (SD)	15.9 (11.3)	16.2 (11.3)	14.4 (11.3)	0.091 ^e^	0.16
**Sensory phenomena**					
Presence	615 (65.6%)	578 (70.6%)	37 (30.6%)	**<0.001**	**5.49 (3.56–8.47)**
Current total score—mean (SD) ^a^	4.9 (4.6)	5.3 (4.6)	2.2 (4.0)	**<0.001 ^f^**	**0.67**
Worst phase total score—mean (SD) ^b^	5.29 (5.0)	5.7 (5.0)	2.4 (4.4)	**<0.001 ^f^**	**0.66**
**Level of insight (BABSscore)**—mean (SD) ^c^	6.8 (5.5)	7.0 (5.5)	5.7 (5.0)	**0.015 ^f^**	**0.24**
**Poor insight**	118 (11.8%)	104 (12.0%)	14 (10.6%)	0.651	1.15 (0.63–2.07)
Insidious onset of OCD symptoms					
Obsessions	694 (68.8%)	612 (70.4%)	77 (58.3%)	**0.005**	**1.70 (1.17–2.48)**
Compulsions	694 (69.3%)	622 (71.6%)	72 (54.6%)	**<0.001**	**2.10 (1.44–3.06)**
**Clinical course**					
Chronic/continuous	62 (6.2%)	54 (6.2%)	8 (6.1%)	0.946	1.03 (0.48–2.21)
Fluctuating (waxing and waning)	313 (31.3%)	270 (31.1%)	43 (32.6%)	0.728	0.93 (0.63–1.38)
Episodic	871 (87.0%)	758 (87.2%)	113 (85.6%)	0.606	1.15 (0.68–1.94)
Worsening with *plateau*	386 (38.6%)	336 (38.7%)	50 (37.9%)	0.863	1.03 (0.71–1.51)
**OCD clinical global impression**					
Mild severity	141 (14.1%)	117 (13.5%)	24 (18.2%)	0.295	1.21 (0.94–1.57)
Moderate severity	336 (33.6%)	291 (33.5%)	45 (34.1%)		
Severe or extremely severe	524 (52.4%)	461 (53.1%)	63 (47.7%)		
Family history of OCS	503 (50.0%)	446 (51.4%)	57 (43.2%)	0.079	1.39 (0.96–2.01)
History of traumatic experiences	677 (67.6%)	593 (68.2%)	84 (63.6%)	0.292	1.23 (0.84–1.80)
History of delayed neuro-psychomotor development	109 (11.2%)	101 (12.1%)	8 (6.2%)	0.052	2.06 (0.98–4.36)
**Suicidality (lifetime)** ^d^					
Already thought life was not worth living	569 (59.3%)	507 (60.9%)	62 (49.2%)	**0.013**	**1.61 (1.10–2.34)**
Already wished to be dead	438 (45.7%)	391 (46.9%)	47 (37.3%)	**0.043**	**1.49 (1.01–2.19)**
Already had suicidal thoughts	348 (36.3%)	313 (37.6%)	35 (27.8%)	**0.033**	**1.57 (1.03–2.37)**
Already planned how to kill him(her)self	199 (20.7%)	181 (21.7%)	18 (14.3%)	0.055	1.67 (0.98–2.82)
Already attempted suicide	104 (10.8%)	98 (11.8%)	6 (4.8%)	**0.018**	**2.67 (1.14–6.24)**
Current suicidal thoughts	104 (10.8%)	93 (11.2%)	11 (8.7%)	0.413	1.31 (0.68–2.53)

Bold values represent the statistically significant results. Underlined variables were included in the logistic regression analysis; OCD: obsessive–compulsive disorder; SD: standard deviation; BDI: Beck Depression Inventory; BAI: Beck Anxiety Inventory; SYM: symmetry; ^a^ 20 missing values; ^b^ 97 missing values; ^c^ 63 missing values; ^d^ 42 missing values; ^e^ Student *t*-test; ^f^ Mann–Whitney test.

**Table 3:** Lifetime comorbid disorders of 1001 OCD patients with (OCD + SYM) and without (OCD − SYM) symptoms of the symmetry dimension.

	Total *n* = 1001	OCD + SYM *n* = 869 (86.8%)	OCD − SYM *n* = 132 (13.2%)	*p*	OR (95% CI)
Any comorbid disorder	881 (88.0%)	770 (88.6%)	111 (84.1%)	0.137	1.47 (0.88–2.46)
Any mood disorder	609 (60.8%)	541 (62.3%)	68 (51.5%)	**0.018**	**1.55 (1.07–2.25)**
Major depressive disorder	565 (56.4%)	503 (57.9%)	62 (47.0%)	**0.018**	**1.55 (1.07–2.24)**
Dysthymia	119 (11.9%)	106 (12.2%)	13 (9.9%)	0.437	1.27 (0.69–2.34)
Bipolar I disorder	38 (3.8%)	36 (4.1%)	2 (1.5%)	0.141	2.81 (0.67–11.83)
Bipolar II disorder	44 (4.4%)	40 (4.6%)	4 (3.0%)	0.412	1.54 (0.54–4.39)
Any anxiety disorder	699 (69.8%)	616 (70.9%)	83 (62.9%)	0.062	1.44 (0.98–2.11)
Separation anxiety disorder	276 (27.6%)	246 (28.3%)	30 (22.7%)	0.181	1.34 (0.87–2.07)
Generalized anxiety disorder	343 (34.3%)	307 (35.3%)	36 (27.3%)	0.069	1.46 (0.97–2.19)
Social anxiety disorder	346 (34.6%)	302 (34.8%)	44 (33.3%)	0.749	1.07 (0.72–1.57)
Specific phobia	314 (31.4%)	272 (31.3%)	42 (31.8%)	0.905	0.98 (0.66–1.45)
Panic disorder/Agoraphobia	202 (20.2%)	179 (20.6%)	23 (17.4%)	0.397	1.23 (0.76–1.99)
Posttraumatic stress disorder	191 (19.1%)	175 (20.1%)	16 (12.1%)	**0.029**	**1.83 (1.05–3.17)**
Any impulse control disorder	362 (36.2%)	323 (37.2%)	39 (29.6%)	0.089	1.41 (0.95–2.10)
Compulsive buying	108 (10.8%)	96 (11.1%)	12 (9.1%)	0.500	1.24 (0.66–2.33)
Skin picking	167 (16.7%)	150 (17.3%)	17 (12.9%)	0.208	1.41 (0.82–2.42)
Trichotillomania	60 (6.0%)	52 (6.0%)	8 (6.1%)	0.972	0.99 (0.46–2.13)
Kleptomania	28 (2.8%)	25 (2.9%)	3 (2.3%)	0.695	1.27 (0.38–4.28)
Intermittent explosive disorder	75 (7.5%)	68 (7.8%)	7 (5.3%)	0.305	1.52 (0.68–3.38)
Any somatoform disorder	175 (17.5%)	155 (17.8%)	20 (15.2%)	0.449	1.22 (0.73–2.02)
Hypochondriasis	34 (3.4%)	28 (3.2%)	6 (4.6%)	0.434	0.70 (0.28–1.72)
Body dysmorphic disorder	117 (11.7%)	107 (12.3%)	10 (7.6%)	**0.114**	**1.71 (1.87–3.37)**
Any eating disorder	114 (11.4%)	101 (11.6%)	13 (9.9%)	0.550	1.20 (0.65–2.21)
Anorexia nervosa	26 (2.6%)	23 (2.7%)	3 (2.3%)	0.801	1.17 (0.35–3.95)
Bulimia nervosa	27 (2.7%)	23 (2.7%)	4 (3.0%)	0.800	0.87 (0.30–2.56)
Binge eating disorder	80 (8.0%)	73 (8.4%)	7 (5.3%)	0.221	1.64 (0.74–3.64)
Alcohol use disorder	79 (7.9%)	70 (8.1%)	9 (6.8%)	0.623	1.20 (0.58–2.46)
Drug use disorder	35 (3.5%)	28 (3.2%)	7 (5.3%)	0.225	0.59 (0.25–1.39)
ADHD *	137 (13.7%)	128 (14.7%)	9 (6.8%)	**0.014**	**2.36 (1.17–4.78)**
Tic disorder	284 (28.4%)	258 (29.7%)	26 (19.7%)	**0.018**	**1.72 (1.09–2.71)**
Tourette syndrome	88 (8.8%)	80 (9.2%)	8 (6.1%)	0.234	1.57 (0.74–3.33)

Bold values represent the statistically significant results. Underlined variables were included in the logistic regression analysis^;^ * attention deficit and hyperactivity disorder; OR: odds ratio; CI: confidence interval; SYM: symmetry.

**Table 4:** Variables that remained associated with the symmetry dimension in the logistic regression.

	Adjusted OR (95% CI)	*z*	*p*
Age at OCS onset	0.92 (0.90–0.95)	−6.17	<0.001
Insidious onset of compulsions	2.01 (1.31–3.10)	3.19	0.001
Depressive symptoms severity (BDI score)	1.02 (1.00–1.04)	2.15	0.031
Sensory phenomena	4.76 (3.07–7.38)	6.99	<0.001

OCS: obsessive–compulsive symptoms; BDI: Beck Depression Inventory; OR: odds ratio; CI: confidence interval.

**Table 5:** Studies describing the frequency of symmetry-related symptoms in OCD patients according to the Y–BOCS or CY–BOCS symptom checklists.

Author, Year, Country	Sample Size and Age	Symmetry	Ordering	Repeating	Counting
Mataix-Cols et al., 1999, USA [37]	354 Adults	44.9%	34.2%	49.7%	35.3%
Cavallini et al., 2002, Italy [68]	180 Adults	31.7%	25.0%	57.8%	16.1%
Hasler et al., 2007, USA [41]	418 Children and adults (≥7 years)	5.9%	47.6%	5.7%	33.5%
Pinto et al., 2007, USA [69]	293 Adults	47.8%	43.3%	56.3%	25.9%
Stein et al., 2007, South Africa [27]	434 Adults	55.8%	36.6%	54.4%	25.4%
Matsunaga et al., 2008, Japan [9]	343 Adolescents and adults (≥ 15 years)	42.0%	22.0%	31.0%	14.0%
Torresan et al., 2009, Brazil [29]	330 Children and adults (10–72 years)	60.3%	49.7%	70.9%	33.6%
van den Heuvel et al., 2009, Netherlands [19]	47 Adults	53.0%	45.0%	68.0%	38.0%
Elvish et al., 2010, England [45]	94 Adults	25.5%	30.9%	26.6%	11.7%
Matsunaga et al., 2010, Japan [32]	343 Adolescents and adults (≥15 years)	42.0%	22.0%	31.0%	14.0%
Prabhu et al., 2013, India [34]	161 Adults	21.1%	23.6%	55.9%	19.3%
Zhang et al., 2013, China [35]	512 Children and adults	64.0%	52.0%	43.0%	27.0%
Asadi et al., 2016, Iran [67]	216 Adults (18–65 years)	47.7%	49.1%	45.1%	31.9%

## Data Availability

The data presented in this study can be available on request from the corresponding author. The data are not publicly available due to ethical reasons.
